# Species identity influences belowground arthropod assemblages via functional traits

**DOI:** 10.1093/aobpla/plt049

**Published:** 2013-10-31

**Authors:** Courtney E. Gorman, Quentin D. Read, Michael E. Van Nuland, Jessica A. M. Bryant, Jessica N. Welch, Joseph T. Altobelli, Morgan J. Douglas, Mark A. Genung, Elliot N. Haag, Devin N. Jones, Hannah E. Long, Adam D. Wilburn, Jennifer A. Schweitzer, Joseph K. Bailey

**Affiliations:** Department of Ecology and Evolutionary Biology, University of Tennessee, Knoxville, TN 37996, USA

**Keywords:** Belowground processes, community similarity, functional plant traits, soil macroinvertebrates, soils, species identity.

## Abstract

Plant species influence belowground communities in a variety of ways, ultimately impacting nutrient cycling. Functional plant traits provide a means whereby species identity can influence belowground community interactions, but little work has examined whether species identity influences belowground community processes when correcting for evolutionary history. Specifically, we hypothesized that closely related species would exhibit (i) more similar leaf and root functional traits than more distantly related species, and (ii) more similar associated soil arthropod communities. We found that after correcting for evolutionary history, tree species identity influenced belowground arthropod communities through plant functional traits. These data suggest that plant species structure may be an important predictor in shaping associated soil arthropod communities and further suggest the importance of better understanding the extended consequences of evolutionary history on ecological processes, as similarity in traits may not always reflect similar ecology.

## Abstract (precis)

Plants link above- and belowground subsystems, and our results suggest that their phylogenetic relationships leave a “fingerprint” on belowground communities. We found that after correcting for evolutionary history, tree species identity influenced belowground arthropod communities through plant functional traits. These data suggest that plant species structure may be an important predictor in shaping associated soil arthropod communities and further suggest the importance of better understanding the extended consequences of evolutionary history on ecological processes, as similarity in traits may not always reflect similar ecology.

## Introduction

Global biodiversity loss is occurring at unprecedented rates (Pimm *et al*. 1995; Sala *et al*. 2000) in response to a variety of human alterations to the environment (Vitousek 1994; Vitousek *et al*. 1997; Chapin *et al*. 2000), making understanding the consequences of such loss on community and ecosystem function a top priority. Much attention has been given to understanding the effects of biodiversity aboveground, with particular emphasis on the relationship between species diversity and primary productivity (Tilman 1996; Hooper and Vitousek 1997; Hector *et al*. 1999; Tilman *et al*. 2001). While these studies have undeniably improved our understanding of the effects of biodiversity, it is equally important to consider how aboveground biodiversity affects the diversity and function of belowground communities, and to understand how above- and belowground communities interact to influence community and ecosystem processes. Interest in the effects of plant species diversity on belowground soil organisms and the soil food web is growing (Kowalchuk *et al*. 2002; Wardle *et al*. 2003; De Deyn *et al*. 2004; Eisenhauer *et al*. 2010); however, the interactions among plant species identity and diversity and belowground communities are not well understood (Wardle 2002; Hooper *et al*. 2005). Plant species influence belowground communities in a variety of ways including the amount of organic matter returned to the soil and the chemical composition of litter (Wardle 2002; Wardle *et al*. 2004), ultimately impacting nutrient cycling. Further understanding of the relationships between species identity and diversity aboveground and community properties and processes belowground is needed to fully understand the consequences of biodiversity loss and to identify the mechanisms of diversity effects.

Functional plant traits provide a means whereby aboveground processes can influence belowground interactions. For example, species-level differences in leaf and root functional traits strongly influence the quality of plant litter inputs to the belowground subsystem, which subsequently impacts microbial communities and associated food web dynamics (Wardle and Lavelle 1997). Using a plant removal design in a grassland system, Wardle *et al*. (1999) found that while larger soil-decomposing animals (i.e. earthworms) were not affected by plant community composition, there were significant responses to the removal treatments at finer taxonomic levels. These organisms were presumably responding to shifts in functional traits rather than species composition, per se. Additionally, tree species in species-rich temperate and tropical forests may possess distinct soil faunal communities (Kaneko *et al*. 2005; Donoso *et al*. 2010; Novotny *et al*. 2010), despite the homogenizing effect of decomposition processes on the soil and litter environment. For example, Donoso *et al*. (2010) found that 12.5–33.3 % of focal species were specialists on certain tree species, presumably due to variation in the ability of tree species to modify leaf litter through differences in functional traits. Thus, aboveground–belowground connections are common; however, the relationships among plant functional traits, plant species identity and soil biota remain unclear.

Using long-term common gardens planted with a variety of eastern North American tree species, our study aims to determine whether tree species identity has ecological consequences for associated belowground communities when correcting for evolutionary history, and to identify the role of plant functional traits as a mechanism for driving soil community differences. Specifically, we hypothesized that after correcting for evolutionary history, closely related species would exhibit (i) more similar leaf and root functional traits than more distantly related species, and (ii) more similar associated soil arthropod communities. We measured plant functional traits above- and belowground, and characterized soil arthropod communities of five tree species that are widespread across the eastern United States. Our results indicate that when correcting for evolutionary history, species identity influences belowground arthropod communities via functional similarity. We speculate that phylogenetic conservatism of functional traits may be playing a role in determining belowground arthropod community assemblages.

## Methods

### Study site and field sampling

To determine whether tree species identity governs functional traits and associated belowground community composition, we studied monocultures of five tree species located at Norris Dam State Park, Tennessee, USA (36.23960°N, 84.10944°W). On a floodplain adjoining the Clinch River, the Tennessee Valley Authority (TVA) previously established experimental forestry plots of several native trees for a hardwood tree improvement programme in the 1960s (specific details unknown by TVA). The plots consisted of ∼25–50 trees per species with trees spaced equally every 3 m; additional woody species were not present in the plots. Plots are underlain by cherty silt loam (Natural Resources Conservation Service (NRCS) Web Soil Survey) and are arranged randomly along the adjacent riparian area (∼45 m from the river). The tree species we sampled included *Quercus alba* (white oak), *Quercus prinus* (chestnut oak), *Juglans nigra* (black walnut), *Ilex opaca* (American holly) and *Liriodendron tulipifera* (tulip-poplar). These species represent three plant orders (Magnoliales, Fagales and Aquifoliales) and four families (Aquifoliaceae, Fagaceae, Juglandaceae and Magnoliaceae) with varying degrees of relatedness.

### Plant functional traits and soil communities and processes

To examine the hypothesis that after correcting for evolutionary history, more closely related tree species had similar functional traits and soil communities, five randomly chosen individual trees were sampled from each plot. Three randomly selected and fully expanded leaves from terminal shoots at the mid-canopy level were collected with pole pruners, and ∼25 cm of root within a 50-cm radius around each tree were collected and stored at 4 °C until analysis. We measured two different functional traits: specific leaf area (SLA) and specific root area (SRA). Specific leaf area is an indicator of potential relative growth rate, gives an indication of investment in leaf structural defence, and typically correlates positively with resource availability (Cornelissen *et al*. 2003). Specific root area is strongly correlated with absorptive activity by the root biomass (Cornelissen *et al*. 2003). To determine SLA (foliar area : mass ratio), leaf area was measured via WinFOLIA (Regent Instruments, Toronto, Canada) and leaves were oven dried at 70 °F for 48 h and the oven-dried leaf mass was recorded. To determine SRA, roots were rinsed with deionized water and then scanned for root surface area and root volume via WinFOLIA software (Regent Instruments). To account for differences in root size between species, we calculated the SRA by dividing the root surface area by root volume.

To characterize soil communities, we collected two 5-cm-diameter soil core samples from the organic layer (unconsolidated organic matter mixed with mineral soil). Soil cores (to a depth of 10 cm) were taken from two opposite sides of the tree ∼1 m from the base of each focal tree. Soil pH (measured in deionized water with a Denver Instruments model 220 pH meter) and soil temperature (soil thermometer at 15 cm depth) were also recorded; however, temperature did not vary among species plots (15.5 °C). The collected soil was stored in a cooler during transport and at 4 °C until analysis. Soils were then sieved (4 mm mesh). The soil from one core was used to determine extracellular enzyme activity and total carbon (C) while the other was used to analyse soil arthropod communities. Arthropods were extracted from soil over 72 h using the Berlese funnel method (Macfadyen 1961). We used a dissection microscope to classify arthropods to order.

We measured potential extracellular enzyme activity of C-degrading enzymes in soil to assess soil quality and microbial activity within the belowground communities. Approximately 1 g of soil, sieved to 2 mm, was analysed for potential activity of α-glucosidase (EC 3.2.1.20) and β-glucosidase (EC 3.2.1.21); 1.0 g of soil was extracted with 50 mmol L^−1^ sodium acetate buffer, pH 6. Both extracellular enzymes are secreted by soil microbes, and can be used as indicators of soil quality; α-glucosidase degrades starch and β-glucosidase oxidizes cellulose. We used methyl-umbelliferone (MUB) as a fluorometric substrate in eight analytical replicates that were incubated for 2 h each; activity was measured on a Synergy HT microplate reader (Sinsabaugh 1994; BioTek Instruments, Inc., Winooski, VT, USA). Potential α-glucosidase enzyme activity levels >100 nmol g^−1^ h^−1^ were discarded, as they were extreme outliers.

Organic matter was removed by the loss-on-ignition technique (Ball 1964) by ashing in a muffle furnace at 550 °C for at least 6 h. Organic matter is measured as the difference in mass before and after combustion in the muffle furnace. Soil organic carbon (SOC) was calculated as 28.4 % of organic matter (Donkin 1991). A subsample of each soil was also oven dried at 105 °C for 48 h to determine soil water content; all final data are reported on an oven-dried mass basis.

### Statistical analyses

We used a phylogeny with branch lengths based on a neutral molecular clock for the five tree species (J. Beaulieu, unpubl. data) to create a pairwise phylogenetic distance matrix (R 2.14.1, ape package). We generated a pairwise distance matrix of the species means of SLA and SRA of the trees. We also generated a pairwise dissimilarity matrix for the soil arthropod community associated with each tree species in monoculture by aggregating the arthropod communities by host tree species, doubly standardizing the aggregated values using Wisconsin standardization, and then calculating the Bray–Curtis dissimilarity between each pair of communities (R 2.14.1, vegan package). Finally, we calculated the pairwise distance between the means of each of the soil properties of each tree species (α-glucosidase, β-glucosidase, total SOC and pH).

We ran Mantel autocorrelation tests with 9999 permutations (R 2.14.1, ade4 package) to determine whether closely related species shared more similar functional trait values than expected by chance and to determine whether pairwise phylogenetic distances between tree species were correlated with either arthropod community similarity or soil properties. In addition, we calculated the Mantel correlation between all pairs of soil property and functional trait distance matrices, including soil pH, SOC, SLA and SRA.

To infer potential mechanisms for the effect of host tree phylogeny on soil arthropod communities, we employed a structural equation modelling approach (Fox 2006; Grace 2006). We used Mantel correlations between pairwise matrices of host tree phylogenetic distances, pairwise root and leaf trait distances, and soil arthropod community Bray–Curtis dissimilarities to create a correlation matrix, which we used to estimate the parameters of the structural equation models that we specified, following the procedure of Leduc *et al*. (1992) and Meneses *et al*. (2012). We selected the models with the lowest Akaike's corrected (AICc) and Bayesian (BIC) information criteria. Modelling was done in R 2.14.1 (sem package).

## Results

### Effects of tree species identity on plant functional traits

Specific leaf area varied roughly 3-fold across the five tree species; *I. opaca* had the thickest, densest leaves, while *L. tulipifera* had the thinnest (Table 1). Specific root area varied slightly less than 2-fold across the sampled species. Again, *I. opaca* and *L. tulipifera* represented the extremes of the continuum, but with *I. opaca* having the most root area (Table 1). Consistent with the expectation that closely related species tend to be more similar functionally, both SLA (*r* = 0.63, *P* = 0.09) and SRA (*r* = 0.71, *P* = 0.06) were conserved across the tree phylogeny, although neither trend was significant at the *α* = 0.05 level.

**Table 1.** Mean values for SLA, SRA, soil pH, SOC, α-glucosidase potential activity (α-GLUC) and β-glucosidase potential activity (β-GLUC) with standard deviations in parentheses.

Species	SLA (cm^2^ g^−1^)	SRA (cm^2^ cm^−3^)	Soil pH	SOC	α-GLUC	β-GLUC
*I. opaca*	65.8 (1.1)	54.6 (12.6)	5.6 (0.2)	2.2 (0.3)	−0.2 (1.3)	48.5 (26.8)
*Q. alba*	154.1 (11.2)	38.9 (6.6)	6.5 (0.2)	2.4 (0.3)	2.7 (2.4)	398.4 (162.4)
*Q. prinus*	126.6 (28.7)	44.1 (8.9)	6.1 (0.4)	2.8 (0.3)	145.3 (230.5)	278.4 (117.3)
*J. nigra*	146.6 (23.8)	37.5 (7.4)	6.5 (0.2)	2.8 (0.5)	116.1 (267.3)	636.8 (160.3)
*L. tulipifera*	177.7 (18.9)	28.6 (7.3)	6.5 (0.2)	2.9 (0.5)	3.4 (1.5)	335.7 (96.3)

### Effects of tree species identity on belowground arthropod communities

**Figure 1.**
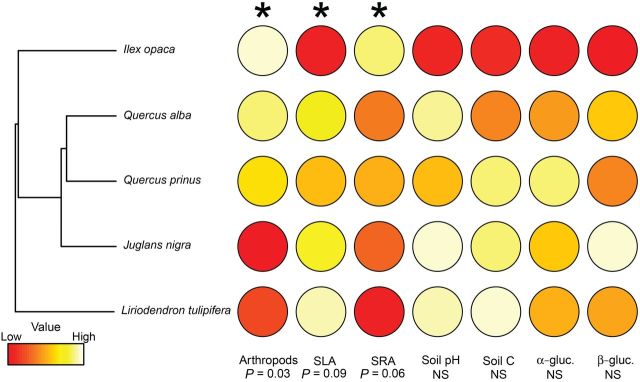
Phylogenetic tree of focal species. Phylogeny of the five focal tree species, with circles shaded by the *Z*-scores **[see Supporting Information]** for soil arthropod community composition nonmetric multidimensional scaling axis 1, host tree SLA and SRA, soil pH, soil organic carbon, and potential α-glucosidase and β-glucosidase activity. The gradient of colours represents the relative magnitude of differences in mean values for measured traits. Significance (*P* values) for Mantel tests between the phylogenetic distance matrix and each trait or soil property distance matrix is given below the figure, with correlations significant at *α* = 0.10 indicated with asterisks.

Six orders of arthropods were identified among all soil samples **[see** **Supporting Information****]**. As would be expected if trait conservatism from associated plants persists in the soil, tree species that had similar functional traits tended to have more similarly structured soil arthropod communities (*r* = 0.58, *P* = 0.03, Fig. 1, Table 2). The two oaks, *Q. alba* and *Q. prinus*, and the walnut *J. nigra* were associated with relatively even soil communities with roughly equal numbers of mites and collembolans. The soil communities underneath *I. opaca* and *L. tulipifera* were both dominated by mites, but *L. tulipifera* hosted a more species-poor community with only two orders represented. Soil pH (range = 0.9) and SOC (range = 0.8 %) were somewhat variable among tree species. Although host tree phylogenetic distance did not predict similarity in pH or SOC in the soil surrounding the trees (*P* > 0.10 in both cases), soils with more similar arthropod communities tended to have more similar pH (*r* = 0.54, *P* = 0.10) and SOC (*r* = 0.62, *P* = 0.05). While potential C-degrading enzyme activity differed widely among soils associated with different tree species, neither α-glucosidase (*r* = 0.30, *P* = 0.36) nor β-glucosidase potential activity (*r* = 0.10, *P* = 0.52) were significantly conserved by host tree species. This is an indication that the effect of tree species identity may become too diffuse to detect at higher levels of organization, and is consistent with our expectations and previous work across levels of organization (Bailey *et al*. 2009).

**Table 2.** Observed Mantel correlations between tree phylogenetic distance (PD), soil invertebrate community composition (IC), and soil (soil pH and soil organic C) and plant properties (SLA and SRA). Bold type indicates a correlation significant at *α* = 0.10, and bold italic type indicates a correlation significant at *α* = 0.05.

	IC	Soil pH	Soil C	SLA	SRA
PD	***0.584***	0.299	0.289	**0.629**	**0.709**
IC		**0.538**	***0.624***	**0.691**	***0.807***
Soil pH			0.601	***0.884***	***0.754***
Soil C				**0.717**	**0.588**
SLA					***0.925***

### Trait-mediated linkages of tree species and arthropod communities

**Figure 2.**
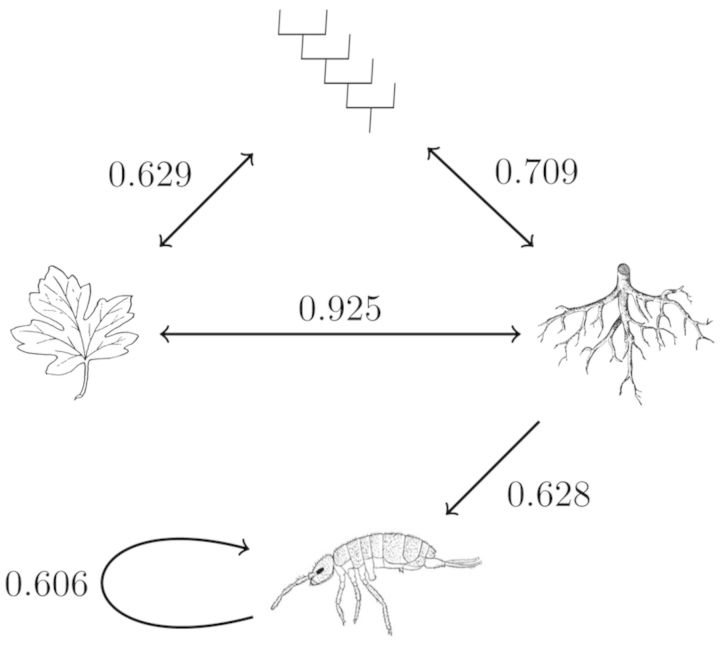
Path diagram of the best structural equation model with bidirectional arrows indicating covariance among host tree phylogeny (branched tree), SLA (leaf) and SRA (root). Unidirectional arrows indicate the estimated effect of tree SRA on soil arthropod community composition (springtail), with the looped path indicating error variance of community composition. Arrows are labelled with the appropriate path coefficient.

Structural equation modelling suggested that the significant effect of host tree species on soil arthropod communities is mediated by SRA, but not SLA (arthropod community *R*^2^ = 0.39, Fig. 2). The best model identified by both AICc and BIC **[see** **Supporting Information****]** did not include any significant residual (i.e. not trait mediated) effect of host tree phylogeny on arthropod community composition. However, it should be noted that only two significantly correlated traits were used in the model, and it is possible that an unmeasured but correlated trait or set of traits may be driving the observed patterns.

## Discussion

Our results demonstrate that after evolutionary history is accounted for, individual tree species support similar communities through conservatism of functional traits. We show that closely related tree species trend toward more similar leaf and root functional traits and soil arthropod communities in comparison to more distantly related species. While previous studies have shown that soil moisture and plot location can affect soil arthropod communities (Luptacik *et al*. 2012), our study controlled these two variables through the use of monoculture plots randomly positioned along a common riparian area with similar soil type and consistent temperature. Our study is the first to our knowledge to examine the effect of host tree identity on soil arthropod community composition, with evolutionary history accounted for; however, similar positive relationships exist for canopy arthropod communities (Novotny *et al*. 2006).

### Trait-mediated plant–soil linkages

Bottom-up forces exert strong control on soil communities through altering resource availability. Detritivores consume dead plant matter and mobilize nutrients, making resources available to the living plant (Setälä 2005). The quantity and quality of organic matter entering the soil subsystem is the primary driver of belowground community structure and function, with fast-growing, short-lived plant tissue promoting bacteria and macroinvertebrates such as earthworms, and slow-growing, long-lived plant tissue promoting fungi and microarthropods such as mites (Wardle *et al*. 2004; Bardgett and Wardle 2010). Because SLA and SRA, traits that are correlated with plant growth rate and patterns of C allocation (Díaz *et al*. 2004), were conserved across species, after accounting for evolutionary history, we expected to see soil properties associated with detrital C processing vary in response to species. Although we found correlations between plant species and the soil community, those effects did not impact soil properties, which could result from the dilution of the effect of litter identity by arthropod processing. Before leaves and other detritus are processed by microbes they are broken down by soil arthropods into smaller fragments to harvest energy and nutrients, causing properties of organic matter originating from different plant species to converge in size and chemistry after it is processed by arthropods (Preston *et al*. 2009; Moore *et al*. 2011). Thus, processing reduces the effect of litter identity, which may be why we found no correlation between C dynamics and tree relatedness. In addition, the lack of observed trait conservatism may be because soil arthropod community composition was analysed at a coarse taxonomic resolution (order), and few tree species were sampled, relative to previous studies (Pokon *et al*. 2005; Novotny *et al*. 2006).

## Conclusions

Plants link above- and belowground subsystems, and their phylogenetic relationships may leave a ‘fingerprint’ on belowground communities. With biodiversity declining rapidly (Pimm *et al*. 1995), it is important to fully understand how species identity aboveground may influence the properties and processes of the belowground system in order to be able to predict how the loss of diversity will affect the functioning of communities and ecosystems. Future studies should particularly focus on functional traits that are conserved across phylogenies. Experimental studies that manipulate tree species identity, with multiple levels of phylogenetic composition or diversity, and measure the response of the soil arthropod community would be especially useful. A mechanistic approach grounded in functional traits and phylogenetic relationships will improve our ability to understand and predict the cascading effects of species loss aboveground on belowground communities and processes. While from a small study, the data reported here suggest important above-/belowground linkages among functional traits and associated communities reflecting past evolutionary history. Studies such as this are critical to bolstering our understanding of the genetic linkages among species and the consequences for community assembly and ecosystem processes.

## Sources of Funding

This work was funded by the Department of Ecology and Evolutionary Biology at the University of Tennessee, Knoxville.

## Contributions by the Authors

C.E.G., Q.D.R., M.E.V., J.A.M.B., M.A.G., J.A.S. and J.K.B. conceived and designed the experiments. C.E.G., Q.D.R., M.E.V., J.A.M.B., J.N.W., J.T.A., M.J.D., E.N.H., D.N.J., H.E.L. and A.D.W. performed the experiments. Q.D.R., C.E.G., M.E.V., J.A.M.B., J.A.S. and J.K.B. analysed the data. C.E.G., Q.D.R., M.E.V., J.A.M.B., J.N.W., J.A.S. and J.K.B. wrote the paper.

## Conflicts of Interest Statement

None declared.

## Supporting Information

The following Supporting Information is available in the online version of this article –

**File 1.** Table. Model selection criteria (ΔAICc and ΔBIC) for the structural equation models. The full model consists of undirected covariances among host tree phylogeny, specific leaf area (SLA) and specific root area (SRA), and directed paths from host tree phylogeny, SLA and SRA to soil invertebrate community composition, as well as error variances for each component.

**File 2.** Table. *Z*-scores for soil community, soil property and functional trait values used to generate Fig. 2.

**File 3.** Table. Number of individuals of each insect order found in each tree treatment.

Additional Information
