# Supplementary material for: Species identity influences belowground arthropod assemblages via functional traits
Source: AoB Plants. 2013 Oct 31;5:plt049. doi: 10.1093/aobpla/plt049 (PMC4104648; doi:10.1093/aobpla/plt049)
Supplement: Additional Information [file supp_5_plt049_index.html]

Species identity influences belowground arthropod assemblages via functional traits — Additional Information 

# Species identity influences belowground arthropod assemblages via functional traits

## Additional Information

Additional Information

**Files in this Data Supplement:**

- Additional Information Table 1 - docx file
- Additional Information Table 2 - docx file
- Additional Information Table 3 - docx file
