# Supplementary material for: Species identity influences belowground arthropod assemblages via functional traits
Source: AoB Plants. 2013 Oct 31;5:plt049. doi: 10.1093/aobpla/plt049 (PMC4104648; doi:10.1093/aobpla/plt049)
Supplement: Additional Information [file supp_plt049_plt049supp_table1.docx]

| **Model description** | **ΔAICc** | **ΔBIC** |
| --- | --- | --- |
| Phylogeny » Invertebrate and SLA » Invertebrate paths removed | 0 | 0 |
| Phylogeny » Invertebrate path removed | 1.02 | 2.11 |
| SLA » Invertebrate path removed | 1.21 | 2.30 |
| SRA » Invertebrate path removed | 2.44 | 3.53 |
| Full model | 3.31 | 4.41 |
