# Supplementary material for: Species identity influences belowground arthropod assemblages via functional traits
Source: AoB Plants. 2013 Oct 31;5:plt049. doi: 10.1093/aobpla/plt049 (PMC4104648; doi:10.1093/aobpla/plt049)
Supplement: Additional Information [file supp_plt049_plt049supp_table2.docx]

| **Tree species** | **Invertebrate NMDS axis 1** | **pH** | **SOC** | **SLA** | **SRA** |
| --- | --- | --- | --- | --- | --- |
| *Q. alba* | 0.797 | 0.579 | -0.631 | 0.470 | -0.194 |
| *Q. prinus* | 0.283 | -0.379 | 0.596 | -0.178 | 0.355 |
| *J. nigra* | -1.261 | 0.721 | 0.596 | 0.293 | -0.340 |
| *I. opaca* | 1.007 | -1.596 | -1.450 | -1.614 | 1.451 |
| *L. tulipifera* | -0.826 | 0.675 | 0.888 | 1.029 | -1.271 |
