# Supplementary material for: Species identity influences belowground arthropod assemblages via functional traits
Source: AoB Plants. 2013 Oct 31;5:plt049. doi: 10.1093/aobpla/plt049 (PMC4104648; doi:10.1093/aobpla/plt049)
Supplement: Additional Information [file supp_plt049_plt049supp_table3.docx]

**File 3**. Table. Number of individuals of each insect order found in each tree treatment.

|  | Acari | Collembola | Coleoptera | Hymenoptera | Diptera | Diplopoda |
| --- | --- | --- | --- | --- | --- | --- |
| *I. opaca* | 18 | 2 | 5 | 0 | 1 | 0 |
| *Q. alba* | 11 | 11 | 1 | 3 | 1 | 1 |
| *Q. prinus* | 11 | 6 | 0 | 1 | 1 | 0 |
| *J. nigra* | 11 | 15 | 2 | 1 | 0 | 0 |
| *L. tulipifera* | 18 | 4 | 0 | 0 | 0 | 0 |
